# Acute caffeine treatment protects the developing retina from ischemia‐induced cell death

**DOI:** 10.1002/2211-5463.70251

**Published:** 2026-05-13

**Authors:** Amanda Alves Nascimento, Danniel Pereira‐Figueiredo, Gabriella Grossi de Lima Dias, Gabriel Ferreira dos Santos, Mariana Rodrigues Pereira, Rafael Brito, Karin C Calaza

**Affiliations:** ^1^ Laboratory Neurobiology of the retina, Biology Institute, Department of Neurobiology and Program of Neurosciences Fluminense Federal University Rio de Janeiro Brazil; ^2^ Laboratory Neurobiology of the retina, Biology Institute, Department of Neurobiology and Program of Biomedical Sciences Fluminense Federal University Rio de Janeiro Brazil; ^3^ Chemical Signaling Laboratory, Biology Institute, Department of Neurobiology, Program of Neurosciences and Program in Science and Biotechnology Fluminense Federal University Rio de Janeiro Brazil; ^4^ Laboratory of Neuronal Physiology and Pathology, Department of Molecular and Cellular Biology, and Graduate Program of Neurosciences, Institute of Biology Fluminense Federal University Rio de Janeiro Brazil

**Keywords:** A_2A_ receptor, Excitotoxicity, Neuroprotection, Oxygen and glucose deprivation

## Abstract

Ischemic damage to the retina during development can lead to irreversible neuronal loss, driven in part by excitotoxic mechanisms and energy deprivation. While two‐day caffeine exposure has previously been shown to confer neuroprotection in this context, it remained unclear whether acute administration during an ischemic event could yield similar benefits. In this study, we used an *ex vivo* model of oxygen and glucose deprivation (OGD) in chick embryo retinas to investigate whether a single, acute dose of caffeine applied during the insult reduces cell death and engages intracellular survival pathways. Results showed a significant reduction in OGD‐induced cytotoxicity by caffeine exposure. However, unlike in the context of chronic exposure, acute caffeine exposure did not increase BDNF expression. Furthermore, antioxidant agents failed to mimic caffeine's protective effects, and acute caffeine exposure did not induce the expression of antioxidant response genes, nor oxidative stress or VEGF expression, suggesting that oxidative stress mitigation is not the primary mechanism for this effect. However, pharmacological antagonism of adenosine A_2A_ receptors with ZM241385 reproduced the neuroprotective effects of caffeine and reduced extracellular glutamate levels during OGD. These findings indicate that acute caffeine administration protects the developing retina primarily through A_2A_ receptor antagonism and inhibition of glutamate excitotoxicity, rather than by activating canonical survival signaling pathways. This supports the potential use of caffeine as a rapid neuroprotective agent during acute ischemic events in the immature central nervous system.

## Graphical abstract

Caffeine reduces cell death in the developing retina under ischemia (OGD). This effect does not involve BDNF upregulation or antioxidant pathways (NRF2/VEGF). Neuroprotection occurs mainly through adenosine A2A receptor antagonism, decreasing glutamate release and excitotoxicity, highlighting caffeine's potential as an acute neuroprotective agent in the immature nervous system.

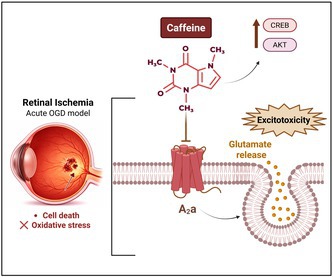

**Abbreviations**
A1: adenosine A1 receptor
A2A: adenosine A2A receptor
AA: ascorbic acid
AKT: protein kinase B
BCA: bicinchoninic acid (assay)
BDNF: brain‐derived neurotrophic factor
CAF: caffeine
cAMP: cyclic adenosine monophosphate
CAT: catalase
cDNA: complementary DNA
CMF: calcium and magnesium‐free
CNS: central nervous system
CO₂: carbon dioxide
CREB: cAMP response element‐binding protein
CTR: control
DMSO: dimethyl sulfoxide
DNA: deoxyribonucleic acid
DNase: deoxyribonuclease
DPCPX: 8‐cyclopentyl‐1,3‐dipropylxanthine (A1 inhibitor)
DTT: dithiothreitol
E16: embryonic day 16
ECL: enhanced chemiluminescence
ERK: extracellular signal‐regulated kinase
GAPDH: glyceraldehyde‐3‐phosphate dehydrogenase
GCL: ganglion cell layer
GSH‐OEt: glutathione monoethyl ester
H₂DCFDA: 2′,7′‐dichlorodihydrofluorescein diacetate
HMOX1: heme oxygenase 1
HRP: horseradish peroxidase
IgG: immunoglobulin G
INL: inner nuclear layer
IPL: inner plexiform layer
LDH: lactate dehydrogenase
MAPK: mitogen‐activated protein kinase
N_2_
: nitrogen
NADH: nicotinamide adenine dinucleotide
NRF2: nuclear factor erythroid 2‐related factor 2
O_2_
: oxygen
OGD: oxygen–glucose deprivation
pAKT: phosphorylated AKT
pCREB: Phosphorylated CREB
pERK: phosphorylated ERK
PI3K: phosphoinositide 3‐kinase
PKA: protein kinase A
RIPA: radio‐immunoprecipitation assay (buffer)
RNA: ribonucleic acid
ROS: reactive oxygen species
RPE: retinal pigment epithelium
RT‐PCR: reverse transcription polymerase chain reaction
SD: standard deviation
SOD1: superoxide dismutase 1
TBS‐T: tris‐buffered saline with Tween 20
Thr202: threonine 202
Tyr204: tyrosine 204
UV: ultraviolet
VEGF: vascular endothelial growth factor
ZM 241385/ZM241385: selective adenosine A2A receptor antagonist

Ischemic damage in the central nervous system (CNS) often leads to irreversible neuronal loss and functional impairment [1]. Among the tissues most vulnerable to this kind of insult is the retina, a highly metabolic tissue of the CNS [2, 3]. Because it operates under tight energy demands, even brief periods of oxygen and glucose deprivation (OGD) can trigger widespread cell death, especially during developmental stages when neural circuits are still being refined [4].

Over the years, a number of neuromodulators have been identified as potential regulators of the retinal response to stress [5, 6, 7]. Adenosine stands out due to its dual role in modulating synaptic transmission and coordinating neuroprotective mechanisms [8, 9, 10]. Depending on which of its four receptors is activated, adenosine can stimulate or inhibit glutamate release and therefore either dampen excitotoxicity or, paradoxically, contribute to tissue vulnerability [11, 12, 13]. Interestingly, caffeine, a widely consumed psychoactive compound, acts as a nonselective antagonist of A_1_ and A_2A_ adenosine receptors, and its neuroprotective properties have drawn attention in models of acute brain injury [14, 15, 16].

In the context of retinal ischemia, most of the available data focus on adult tissues. Far less is known about how the developing retina responds to caffeine under stress. In a previous study, we showed that previous *in vivo* exposure to nontoxic caffeine doses during embryonic development reduced ischemic damage in chick retinas, via modulation of intracellular signaling pathways involved in cell survival, namely AKT, ERK, and CREB [17]. These findings suggested caffeine might induce a preconditioning‐like state in the immature retina [17].

Nevertheless, a critical question was raised: Is the previous exposure necessary, or could an acute dose of caffeine, administered at the moment of insult, also confer neuroprotection? In other words, does the protective effect observed with pre‐exposure occur when caffeine is administered acutely, under conditions in which the tissue is already experiencing stress? Furthermore, we still do not know whether the same molecular pathways are recruited during such acute intervention.

To explore this, we hypothesized that caffeine exposure during an acute ischemic event would be sufficient to protect the developing retina. To test this hypothesis, we used an *ex vivo* model of oxygen and glucose deprivation in chick embryo retinas. Caffeine was administered during the ischemic insult at a final concentration of 140 μm, and we assessed both the extent of retinal cell death and the activation state of key signaling proteins. In addition, we also assessed the involvement of the same intracellular signaling cascades previously associated with preconditioning (namely, CREB, ERK, and AKT activation). Since oxidative stress and hypoxia‐responsive pathways are known contributors to retinal injury during ischemia, we also evaluated the putative antioxidant effect of caffeine. This approach enabled us to evaluate whether acute caffeine exposure is not only protective but also capable of engaging endogenous survival pathways in the absence of prior preconditioning.

## Materials and methods

### Animals

All experimental procedures conducted at the Fluminense Federal University complied with the guidelines established by the Animal Research Ethics Committee (CEPA/Proppi/UFF), under project approval number 197/2012. Fertilized eggs from the White Leghorn strain (*Gallus domesticus*) were obtained from a local farm in Rio de Janeiro, RJ, Brazil. The eggs were incubated manually (Chocadeira Manual, Premium Ecológica, Belo Horizonte, MG, Brazil) at a constant temperature of 37.8 °C, with humidity maintained at 30 °C as measured by a wet‐bulb thermometer until hatching.

Retinas from 16‐day‐old chicken embryos (E16) were collected for oxygen and glucose deprivation experiments, matching the developmental stage previously studied [17]. At E16, key developmental phenomena include the onset of synaptogenesis in the inner plexiform layer (IPL), the completion of retinal cell proliferation, the cessation of natural cell death in the ganglion cell layer (GCL), and its near completion in the inner nuclear layer (INL), as well as the initiation of opsin expression and development of the photoreceptor outer segment [18].

Embryos were randomly selected for *ex vivo* retinal preparations subjected to the OGD protocol. Although these retinas already exhibit functional maturation, sex determination was not performed due to the embryonic age and the absence of visible sexual dimorphism at this developmental stage. Embryo staging was determined according to Hamilton and Hamburger [19].

### Reagents

Caffeine (Cat. C0750; Sigma‐Aldrich, St. Louis, MO, USA) was used at a final concentration of 140 μm to mimic the estimated *in ovo* exposure resulting from chronic administration of 30 mg·kg^−1^. This estimate assumes an approximate embryonic distribution volume of 1 L·kg^−1^ and ~ 50% maternal‐to‐embryo transfer, consistent with prior pharmacokinetic considerations in chick embryos [17]. Antioxidants such as ascorbic acid (AA; Cat. A4544), dithiothreitol (DTT; Cat. D0632), and glutathione ethyl ester (GSH‐OEt; Cat. G1404), all from Sigma‐Aldrich (St. Louis, MO, USA), were prepared at concentrations of 100 μm or 500 μm. Reactive oxygen species were measured using the fluorescent probe 2′,7′‐dichlorodihydrofluorescein diacetate (H₂DCFDA; Invitrogen, Cat. No. C6827).

Adenosine receptor antagonists 8‐cyclopentyl‐1,3‐dipropylxanthine (DPCPX; Cat. D047) and ZM 241385 (Cat. Z0000) (Sigma‐Aldrich, St. Louis, MO, USA) were dissolved in dimethyl sulfoxide (DMSO; Cat. D12345; Invitrogen/Thermo Fisher Scientific, Waltham, MA, USA) and applied at final concentrations of 0.1, 1.0, or 10 μm. The final concentration of DMSO in the incubation medium did not exceed 0.1% (v/v) in any experimental condition and identical DMSO volumes were added to vehicle control samples. Ringer's solution was used as the negative control, while DMSO served as the vehicle control in experiments involving drugs dissolved in DMSO. For protein quantification, the bicinchoninic acid (BCA) assay kit (Cat. 23 227; Invitrogen, Thermo Fisher Scientific, Waltham, MA, USA) was used.

Primary antibodies included anti‐CREB (Cat. 48H2), anti‐phospho‐CREB Ser133 (Cat. S133), anti‐Akt (Cat. C67E7), anti‐phospho‐Akt Ser473 (Cat. D9E), anti‐p44/42 MAPK Erk1/2 (Cat. 137F5), and anti‐phospho‐p44/42 MAPK Erk1/2 Thr202/Tyr204 (Cat. T202/Y204), all from Cell Signaling Technology (Danvers, MA, USA). Anti‐BDNF (Cat. 500‐P84) was obtained from PeproTech (Rocky Hill, NJ, USA). Anti‐catalase (Cat. sc‐271 803; Santa Cruz Biotechnology, Dallas, TX, USA), anti‐NRF2 (Cat. C‐20: sc‐722; Santa Cruz Biotechnology, Dallas, TX, USA), and anti‐VEGF (Cat. 500‐P131; Thermo Fisher Scientific, Waltham, MA, USA) were also used. For loading controls, anti‐α‐tubulin (Cat. 3873; Cell Signaling Technology, Danvers, MA, USA), anti‐β‐actin (Cat. 20–33; Sigma‐Aldrich, St. Louis, MO, USA), and anti‐GAPDH (Cat. 258; Thermo Fisher Scientific, Waltham, MA, USA) were used.

HRP‐conjugated secondary antibodies against rabbit IgG (Cat. NA934; Amersham Pharmacia Biotech, Buckinghamshire, UK) and mouse IgG (Cat. NA931; GE Healthcare, Chicago, IL, USA) were used for detection. Chemiluminescence signal was revealed with the ECL kit (Cat. RPN2232; Amersham Pharmacia Biotech, Buckinghamshire, UK). CytoTox 96^®^ Non‐Radioactive Cytotoxicity Assay colorimetric kit was purchased from Promega (Madison, WI, USA). cOmplete™ Mini cocktail protease inhibitor was obtained from Roche (Germany).

All stock solutions were stored at −20 °C. In vehicle control conditions, the same final volume of DMSO was added as in treated samples. For the OGD protocol, gas mixtures containing 95% O_2_/5% CO_2_ and 95% N_2_/5% CO_2_ were obtained from White Martins, Praxair Inc. (Rio de Janeiro, Brazil). All other reagents were of analytical grade or higher purity.

### OGD in retinas *ex vivo*

Embryos were euthanized by decapitation, and their eyes were enucleated. Each eye was sectioned along the sagittal plane, and the posterior segment (eyeball posterior half containing the neural retina and excluding the anterior chamber and lens) was isolated. Entire retinas were carefully dissected away from the RPE (retinal pigment epithelium) and used as single samples and divided randomly for use in the OGD procedure. After OGD, the incubation medium (extracellular fraction) of each group was collected immediately after the 50‐min incubation (≈ 100 μL per sample) and frozen at −20 °C for later LDH and glutamate analysis.

For the cell viability assessment using LDH activity and for protein analysis by western blotting, the retinas were dissected away from the RPE in a calcium‐ and magnesium‐free (CMF) medium at 37 °C. For the control condition, retinal segments were incubated in Ringer's solution containing 120 mm NaCl, 3 mm KCl, 1 mm NaH_2_PO_4_, 30 mm NaHCO_3_, 1 mm CaCl_2_, 1 mm MgCl_2_·6H_2_O, and 10 mm glucose. In contrast, OGD conditions were induced by incubating retinal tissue in glucose‐free Ringer's solution. Before incubation, both the control and OGD Ringer's solutions were aerated for 15 min: the control solution with a gas mixture of 95% O_2_ and 5% CO_2_, and the OGD solution with 95% N_2_ and 5% CO_2_. The pH of both solutions was adjusted to 7.2–7.4.

Following preparation, retinal segments were incubated in either the control or OGD solution for 50 min. During this period, each solution was continuously and gently bubbled with its respective gas mixture (control: 95% O_2_/5% CO_2_; OGD: 95% N_2_/5% CO_2_) and maintained at 37 °C in a water bath. In some experiments, caffeine (140 μm) was added to the solution and remained present throughout the incubation period, while in others, adenosine receptor antagonists (DPCPX or ZM 241385 at 0.1, 1.0, or 10 μm) were applied instead. The corresponding incubation medium, representing the extracellular fraction, was also collected and frozen to be used for the cell viability assay. For experiments investigating the effects of antioxidants and adenosine receptor antagonists, the drugs were added directly to the OGD solution and remained present throughout the incubation period.

All pharmacological agents used in the study were stored at −20 °C. In experiments without drug treatment, the equivalent volume of the vehicle used to dissolve the drugs was added to maintain consistency across all experimental conditions.

### Western blotting

Following the OGD procedure, retinal segments were collected in RIPA buffer (150 mm NaCl, 50 mm Tris‐base, 5 mm EGTA, 1% Triton X‐100, 0.5% DOC, 0.1% SDS, pH 7.5) supplemented with protease inhibitors and 1 mm DTT. Samples were homogenized, and protein concentrations were determined using the bicinchoninic acid (BCA) method. Supernatants were mixed with denaturing buffer (0.5 M Tris–HCl/0.4% SDS, pH 6.8, 30% glycerol, 10% SDS, 0.6 M DTT, and 0.01% bromophenol blue), heated at 100 °C for 5 min, and stored at −20 °C.

Protein extracts (30 μg per lane) were separated on a 4–10% SDS/polyacrylamide gel and transferred onto PVDF membranes. Membranes were blocked in 5% skimmed milk diluted in Tris‐buffered saline with 0.1% Tween‐20 (TBS‐T) and incubated overnight at 4 °C with the following primary antibodies: CREB (1 : 2000), phospho‐CREB Ser133 (1 : 1000), Akt (1 : 2000), phospho‐Akt Ser473 (1 : 1000), p44/42 MAPK Erk1/2 (1 : 2000), phospho‐p44/42 MAPK Erk1/2 T202/Y204 (1 : 1000), BDNF (1 : 500), NRF2 (1 : 2000), and catalase (1 : 2000). For normalization, α‐tubulin (1 : 100 000), β‐actin (1 : 5000), and GAPDH (1 : 1000) were used as loading controls. As a general approach, α‐tubulin was used as the primary loading control; however, when the molecular weight of the target protein was close to that of α‐tubulin or when primary antibodies from the same species were used, alternative controls were employed to avoid signal overlap. Catalase was normalized to β‐actin due to its similar molecular weight to α‐tubulin (~ 60 vs. ~ 55 kDa) and the use of mouse primary antibodies for both proteins. VEGF was normalized to α‐tubulin rather than β‐actin due to their similar molecular weights (~ 42 kDa). BDNF was normalized to GAPDH because of overlapping molecular weights with α‐tubulin (~ 50 kDa). For phosphorylated proteins (pAKT, pCREB, and pERK), densitometric values were normalized to their respective total protein levels (AKT, CREB, and ERK).

After primary antibody incubation, membranes were washed three times with TBS‐T and incubated with appropriate secondary antibodies. Protein bands were detected using the ECL Prime chemiluminescence system, and images were acquired with the ChemiDoc^TM^ MP Imaging System. Quantification of band intensities was performed using imagej software (version 1.53). Although α‐tubulin expression may be influenced by ischemic conditions, no significant changes in its levels were observed across the groups.

### Real‐time quantitative PCR (RT‐qPCR)

Total RNA was extracted from E16 retinas using Trizol reagent, according to the manufacturer's instructions (Thermo Fisher Scientific, MA, USA). The amount and purity of total RNA were evaluated with a UV spectrophotometer (NanoDrop 2000, Thermo Fisher Scientific, MA, USA) by A260/280 ratio. Extracted RNA samples were treated with RNase‐free DNase (Cat. No: AM1906, Thermo Fisher Scientific, MA, USA). First‐strand synthesis of cDNA was performed using First‐Strand cDNA synthesis kit, according to the manufacturer's instructions (Cat. No: GE27‐9261‐01, Cytiva, MA, USA). Quantitative PCR was performed using GoTaq^®^ qPCR Master Mix (Cat. No: A6002, Promega Corporation, WI, USA) in a StepOne^TM^ Real‐Time PCR System (Thermo Fisher Scientific, MA, USA). The cycling conditions were as follows: initial denaturation at 95 °C for 2 min, followed by 40 cycles of denaturation at 95 °C for 15 s and annealing/extension at 60 °C for 1 min. A melt curve analysis (0.3 °C increment every 20 s up to 95 °C) was performed to confirm amplification specificity.

Species‐specific primers for Gallus gallus were designed using Primer‐BLAST (NCBI) and synthesized by Thermo Fisher Scientific. Primer sequences are listed in Table 1. Relative gene expression was calculated using the 2^‐^^ΔΔCt^ method, with RPL27 as the endogenous reference gene. All reactions were performed in technical triplicates, and at least three independent biological replicates were analyzed per experimental group.

**Table 1:** List of primers.

Genes	Name and accession number	Sequence
NRF2	MN416129.1 Gallus gallus NF‐E2‐related factor 2 (NRF2)	Forward:GGACGGTGACACAGGAACAA
		Reverse:CTCCACAGCGGGAAATCAGA
CAT	NM_001031215.2 Gallus gallus catalase (CAT)	Forward:GTTCTGAAGGAGAGCCGCAT
		Reverse:GTCTCGCACCTGAGACACAT
HMOX1	NM_205344.2 Gallus gallus heme oxygenase 1 (HMOX1)	Forward:GCGGAGAACACACCCTTCAT
		Reverse:GTGACCAGCTTGAACTCGTG
SOD1	NM_205064.2 Gallus gallus superoxide dismutase 1, soluble (SOD1)	Forward:CAGATAGGCACGTGGGTGAC
		Reverse:CAGTGTGGTCCGGTAAGAGA
RPL27	NM_205337.1 Gallus gallus ribosomal protein L27 (RPL27)	Forward:AAGCCGGGGAAGGTGGTG
		Reverse:GGCCGATCAGACGTGCCA

### Measurement of intracellular reactive oxygen species (ROS)

Intracellular reactive oxygen species (ROS) were measured using the fluorescent probe 2′,7′‐dichlorodihydrofluorescein diacetate (H_2_DCFDA). Retinas from E16 chick embryos were dissected and incubated with the probe (10 μm) diluted in oxygenated Ringer–Locke solution for 45 min at 37 °C. Control and OGD‐treated retinas were incubated under the same experimental conditions used in the OGD protocol. After incubation, retinal tissues were washed twice with Ringer solution and further incubated in fresh Ringer solution for 20 min to remove excess probe. Tissues were then lysed in 1% Triton X‐100 and mechanically homogenized. The samples were centrifuged (5000 rpm, 3 min), and the supernatant was collected for fluorescence measurement. Fluorescence intensity was measured using a fluorimeter (Biotek) with excitation at 493 nm and emission at 522 nm.

### Cell viability assessment

Cell viability was evaluated using the CytoTox 96^®^ Non‐Radioactive Cytotoxicity Assay, a colorimetric method based on the quantification of lactate dehydrogenase (LDH) activity. In this assay, LDH released from damaged cells catalyzes the conversion of lactate to pyruvate, generating NADH that drives the reduction in a tetrazolium salt to a red formazan product, which is quantified colorimetrically at 490 nm. Retinal segments were incubated in a lysis buffer at room temperature for 30 min to obtain the intracellular LDH fraction, after which they were stored at −20 °C. Each retina (*n* = biological replicates indicated in figure legends; typically *n* = 3–5) was considered a biological replicate. Measurements were performed in duplicate as technical replicates. After incubation under OGD or normoxic conditions, the extracellular medium (~ 100 μL per sample) was collected to quantify LDH released from damaged cells. This medium was stored at −20 °C and subsequently combined with the corresponding intracellular lysate when total LDH activity was calculated.

Before analysis, both intracellular and extracellular samples were thawed, homogenized, and centrifuged at 2000 × ***g*** (approximately 35.8 G) for 4 min at 25 °C. For LDH quantification, 5 μL of the intracellular sample was mixed with 20 μL of the extracellular sample, and each measurement was performed in duplicate.

The colorimetric signal was read at 490 nm using a microplate reader (iMARK^TM^, Bio‐Rad, Hercules, CA, USA; RRID: SCR_023799). The mean absorbance values for each condition were used to determine LDH activity in both compartments. Total LDH was obtained by summing the intracellular and extracellular values. The percentage of LDH released, an indicator of membrane damage and cytotoxicity, was calculated by dividing the extracellular LDH activity by the total LDH, with appropriate adjustments for sample dilutions.

### Statistical analysis

Statistical analyses were performed using graphpad prism version 10 (GraphPad Software, San Diego, CA, USA). Data are expressed as mean ± standard deviation (SD), obtained from independent biological replicates. Both eyes from each embryo were used in the experiments. After dissection, each retina was divided into two halves, generating four retinal fragments per embryo. These fragments were randomly assigned to the different experimental groups. Each retinal fragment was considered an independent biological replicate (*n* = 3–5 per group). All assays were performed in duplicate as technical replicates. Comparisons involving two experimental factors (e.g., normoxia vs. OGD and pharmacological treatments) were analyzed by two‐way ANOVA, followed by Bonferroni's multiple comparisons test. Statistical significance was defined as *P* < 0.05. Outliers falling outside the 95% confidence interval were excluded from the final analysis.

## Results

**Fig. 1:**
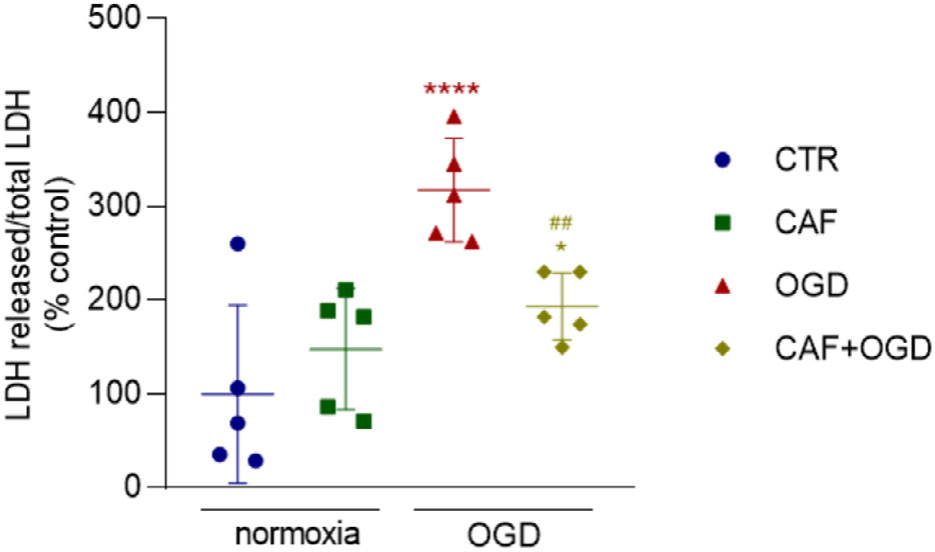
Caffeine reduced OGD‐induced LDH release compared with OGD alone, representing partial but significant protection. Retinal segments from E16 chick embryos were subjected to 50 min of normoxia or oxygen–glucose deprivation (OGD) in the presence or absence of caffeine (140 μm). Cell death was assessed by measuring LDH activity in the extracellular medium per total LDH. Caffeine significantly reduced OGD‐induced LDH release, indicating partial retinal protection. Values are expressed as mean ± standard deviation (SD): Control (100.0 ± 94.7, *n* = 5), Caffeine (147.9 ± 64.3, *n* = 5), OGD (317.5 ± 55.0, *n* = 5), OGD + Caffeine (193.4 ± 35.7, *n* = 5). Two‐way ANOVA revealed a significant effect of condition (F(1,16) = 40.07, *P* < 0.0001) and a significant interaction between treatment and condition (F(1,16) = 17.14, *P* = 0.0008), but no main effect of treatment alone (F(1,16) = 3.36, *P* = 0.0856). Bonferroni's *post hoc* test confirmed significant differences between Control and OGD (****P < 0.0001), Control and OGD + Caffeine (*P < 0.05), and OGD and OGD + Caffeine (**P < 0.01). OGD, oxygen–glucose deprivation; LDH, lactate dehydrogenase; CAF, caffeine.

As introduced earlier, one of our main goals was to evaluate whether caffeine could exert a protective effect against cell death when administered acutely during an ischemic insult. To replicate the estimated *in ovo* concentration of 30 mg·kg^−1^ caffeine used in our previous study, a final concentration of 140 μm was employed in this acute model. In this *ex vivo* ischemia paradigm, caffeine remained in the retinal incubation medium throughout the entire procedure. Strikingly, we observed a significant reduction in OGD‐induced cell death when caffeine was present during the insult, corresponding to an approximate 39% decrease in LDH release relative to untreated OGD (partial protection) (Fig. 1).

**Fig. 2:**
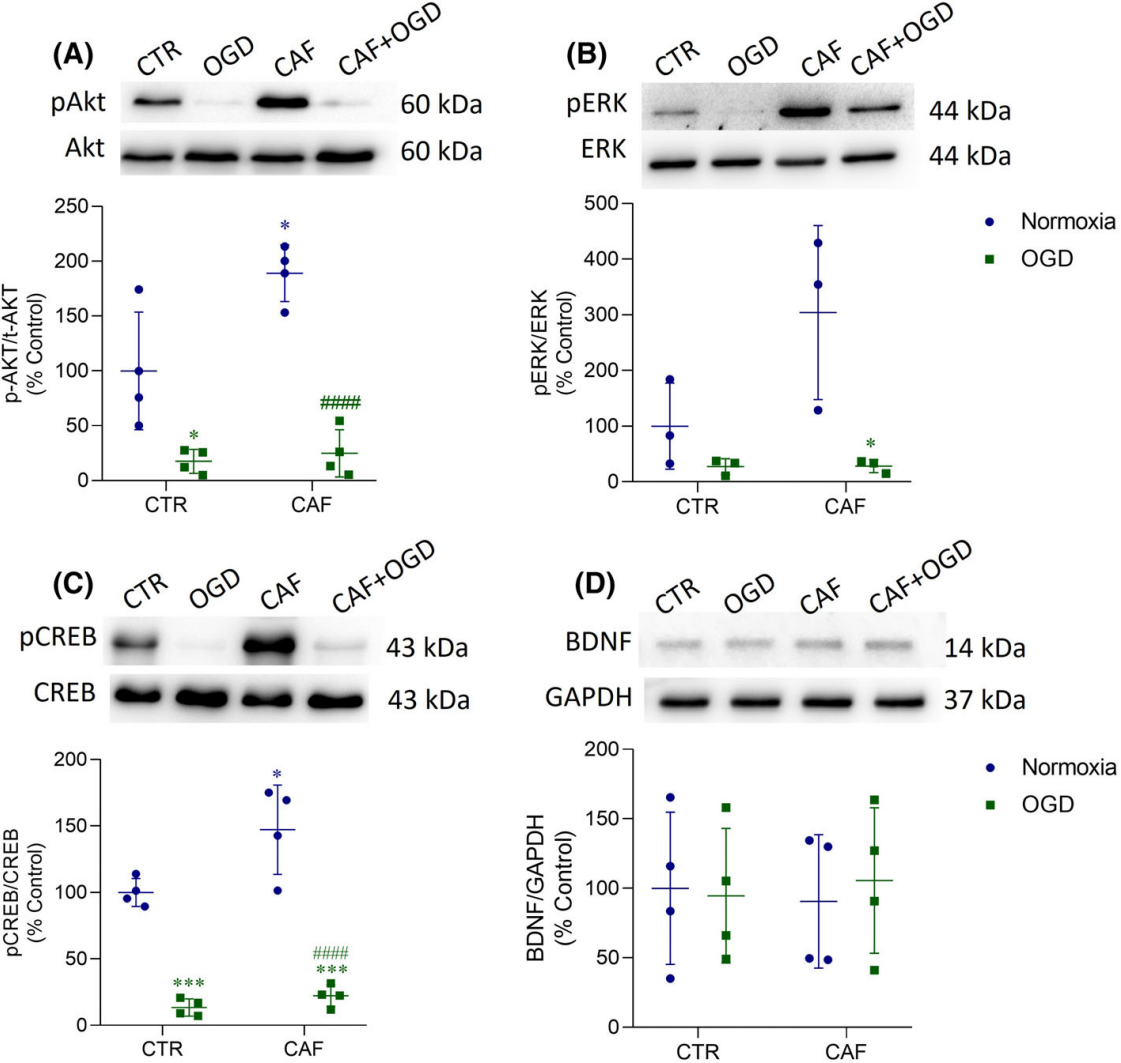
Caffeine increases phosphorylation of Akt, ERK, and CREB under normoxia, but not during OGD. Western blot analysis of retinal tissue exposed to 50 min of normoxia (●) or oxygen–glucose deprivation (OGD; ■), in the presence or absence of caffeine (140 μm), was performed to quantify phosphorylated Akt (pAkt) (A), ERK (pERK) (B), CREB (pCREB) (C), and BDNF (D). (A) pAkt: Control (100.0 ± 53.6, *n* = 4), OGD (17.6 ± 10.9, *n* = 4), caffeine (189.1 ± 25.8, *n* = 4), OGD + caffeine (24.8 ± 21.6, *n* = 4). Two‐way ANOVA revealed significant effects of condition (F(1,12) = 194.3, *P* < 0.0001), treatment (F(1,12) = 29.64, *P* = 0.0001), and their interaction (F(1,12) = 21.36, *P* = 0.0006), indicating that OGD markedly reduced pAkt levels, whereas caffeine increased Akt phosphorylation under normoxia but not during OGD. (B) pERK: Control (100.0 ± 77.1, *n* = 3), OGD (27.2 ± 14.4, *n* = 3), caffeine (304.0 ± 156.3, *n* = 3), OGD + caffeine (28.3 ± 11.7, *n* = 3). Two‐way ANOVA revealed a significant effect of condition (F(1,8) = 14.71, *P* = 0.0050), but no significant effect of treatment (*P* = 0.0540) or interaction (*P* = 0.0561). These results indicate that OGD strongly reduced ERK phosphorylation. Although caffeine increased pERK levels under normoxia, this effect did not reach statistical significance after correction for multiple comparisons. (C) pCREB: Control (100.0 ± 10.5, *n* = 4), OGD (13.5 ± 6.5, *n* = 4), caffeine (147.2 ± 33.6, *n* = 4), OGD + caffeine (22.3 ± 9.8, *n* = 4). Two‐way ANOVA revealed significant effects of condition (F(1,12) = 10.12, *P* = 0.0079), treatment (F(1,12) = 144.3, *P* < 0.0001), and their interaction (F(1,12) = 4.76, *P* = 0.0498). OGD caused a pronounced reduction in CREB phosphorylation, whereas caffeine markedly increased pCREB under normoxia. Notably, despite this strong activation under normoxia, caffeine did not prevent the reduction in CREB phosphorylation induced by OGD. (D) BDNF: Control (100.0 ± 54.8, *n* = 4), OGD (94.6 ± 48.4, *n* = 4), caffeine (90.6 ± 48.0, *n* = 4), OGD + caffeine (105.6 ± 52.3, *n* = 4). Two‐way ANOVA detected no significant effects of condition, treatment, or interaction (all *P* > 0.64), indicating that BDNF levels remained unchanged across all experimental conditions. Values are expressed as mean ± standard deviation (SD). Statistical significance was determined by two‐way ANOVA followed by Bonferroni's multiple comparisons test. *P < 0.05, ***P < 0.001 vs. control (CTR normoxia); #*P* < 0.05, ####*P* < 0.0001 vs. CAF (normoxia with caffeine). OGD, oxygen–glucose deprivation; pAkt, phosphorylated Akt; pERK, phosphorylated ERK; pCREB, phosphorylated CREB; BDNF, brain‐derived neurotrophic factor.

Prompted by this observation, we investigated whether the protective effects observed under acute caffeine treatment involve the same intracellular pathways previously associated with two‐day exposure, particularly the upregulation of signaling proteins and the neurotrophin BDNF. To test this, we examined the levels of phosphorylated Akt (pAkt), ERK (pERK), and CREB (pCREB), as well as BDNF content, in retinas subjected to the same acute caffeine treatment. Interestingly, even in this short timeframe, caffeine increased the phosphorylation of Akt, ERK, and CREB under normoxic conditions. However, BDNF levels did not show significant differences, including those exposed to caffeine and/or OGD (Fig. 2). In addition, the levels of phosphorylated‐Akt, ‐ERK, and ‐CREB significantly reduced in the OGD context (Fig. 2).

**Fig. 3:**
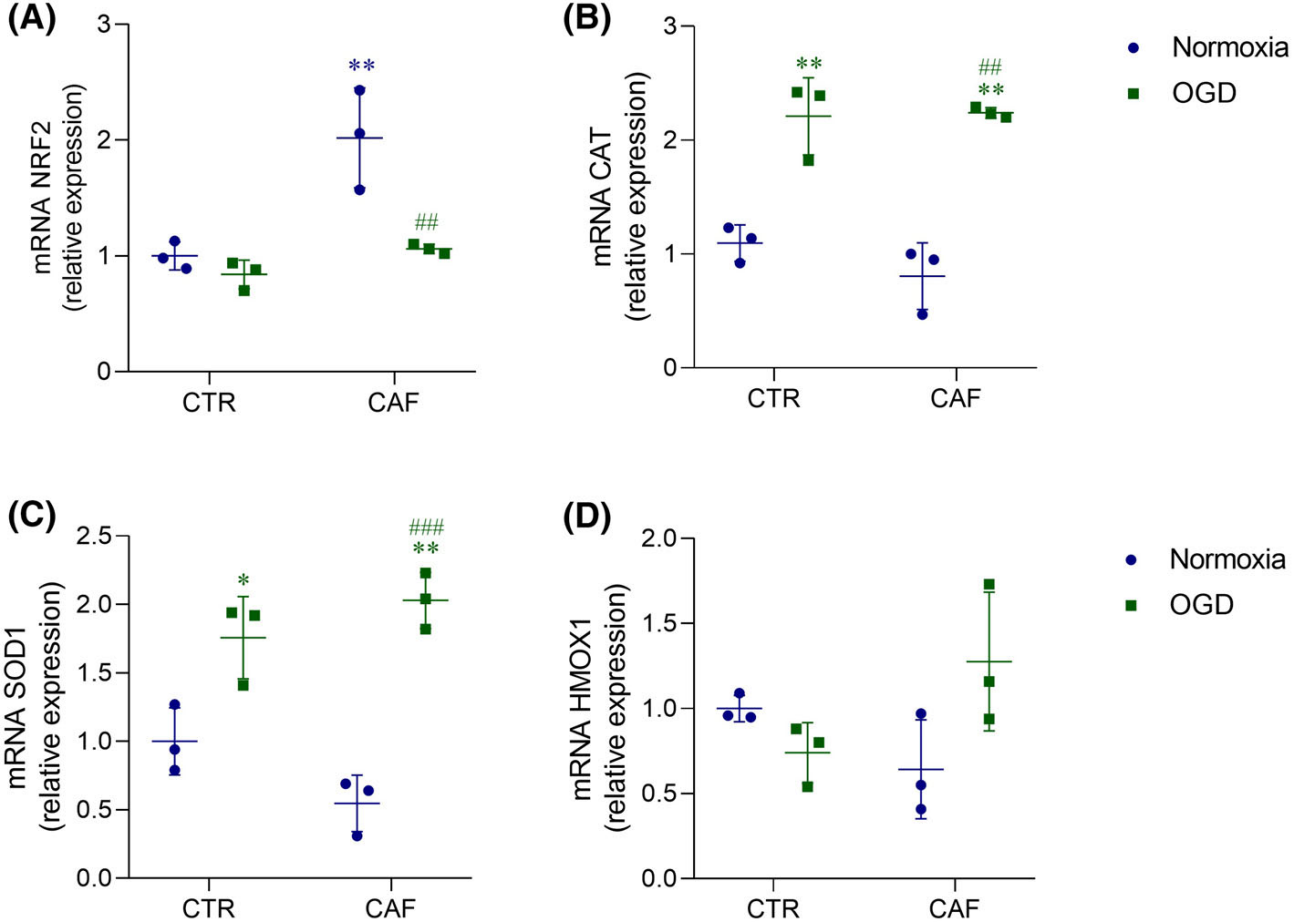
Acute caffeine does not restore NRF2‐dependent antioxidant gene expression during OGD. Retinas were exposed to 50 min of normoxia (●) or OGD (■) in the presence or absence of caffeine (140 μm). Relative mRNA expression of (A) NRF2, (B) catalase (CAT), (C) superoxide dismutase 1 (SOD1), and (D) heme oxygenase‐1 (HMOX1) was quantified by RT‐qPCR. Values are expressed as mean ± SD (*n* = 3). (A) NRF2 – Control (1.000 ± 0.1212), Caffeine (2.019 ± 0.4313), OGD (0.8400 ± 0.1249), Caffeine + OGD (1.060 ± 0.0400). Two‐way ANOVA revealed significant effects of interaction (F(1,8) = 8.797, *P* = 0.0180), treatment (F(1,8) = 21.15, *P* = 0.0018), and condition (F(1,8) = 17.25, *P* = 0.0032). Bonferroni's test showed significant differences between Control and Caffeine (*P* = 0.0041), OGD and Caffeine (*P* = 0.0016), and Caffeine and Caffeine + OGD (*P* = 0.0061). No significant difference was observed between OGD and Caffeine + OGD. (B) CAT – Control (1.097 ± 0.1595), Caffeine (0.8067 ± 0.2926), OGD (2.210 ± 0.3381), Caffeine + OGD (2.240 ± 0.04583). Two‐way ANOVA showed a significant effect of condition (F(1,8) = 85.54, *P* < 0.0001), with no significant interaction or treatment effects. Bonferroni's test indicated significant differences between Control and OGD (*P* = 0.0027), Control and Caffeine + OGD (*P* = 0.0022), OGD and Caffeine (*P* = 0.0006), and Caffeine and Caffeine + OGD (*P* = 0.0005). No difference was detected between OGD and Caffeine + OGD. (C) SOD1 – Control (1.000 ± 0.2456), Caffeine (0.5467 ± 0.2065), OGD (1.757 ± 0.3004), Caffeine + OGD (2.030 ± 0.2052). Two‐way ANOVA revealed significant effects of interaction (F(1,8) = 6.733, *P* = 0.0319) and condition (F(1,8) = 63.98, *P* < 0.0001). Bonferroni's test showed significant differences between Control and OGD (*P* = 0.0305), Control and Caffeine + OGD (*P* = 0.0049), OGD and Caffeine (*P* = 0.0017), and Caffeine and Caffeine + OGD (*P* = 0.0004). No difference was detected between OGD and Caffeine + OGD. (D) HMOX1 – Control (1.000 ± 0.07810), Caffeine (0.6433 ± 0.2914), OGD (0.7400 ± 0.1778), Caffeine + OGD (1.277 ± 0.4077). Two‐way ANOVA revealed a significant interaction effect (F(1,8) = 8.288, *P* = 0.0205), with no significant main effects of treatment or condition. Bonferroni's multiple comparisons did not detect significant pairwise differences between groups. *P* < 0.05, **P < 0.01 vs Control; *P* < 0.05, ##*P* < 0.01 vs Caffeine (normoxia). OGD, oxygen–glucose deprivation; NRF2, nuclear factor erythroid 2‐related factor 2; CAT, catalase; SOD1, superoxide dismutase 1; HMOX1, heme oxygenase‐1.

Given previous reports that caffeine can act as an antioxidant, either by directly scavenging reactive oxygen species or by boosting endogenous antioxidant enzymes [20, 21, 22, 23], we next tested whether caffeine could modulate the endogenous antioxidant machinery at the transcriptional level. The mRNA expression of NRF2 and selected NRF2‐regulated genes (CAT, SOD1, and HMOX1) was evaluated by RT‐qPCR (Fig. 3).

OGD significantly increased the expression of CAT and SOD1 antioxidant‐related genes, indicating activation of a stress‐responsive transcriptional program. Under normoxic conditions, caffeine increased NRF2 expression, but no alteration was detected after OGD (Fig. 3A). However, during OGD, caffeine did not restore or further enhance the expression of CAT, SOD1, or HMOX1 (Fig. 3B–D). Importantly, no differences were detected between CTR‐OGD and CAF‐OGD groups for any of the antioxidant genes analyzed.

**Fig. 4:**
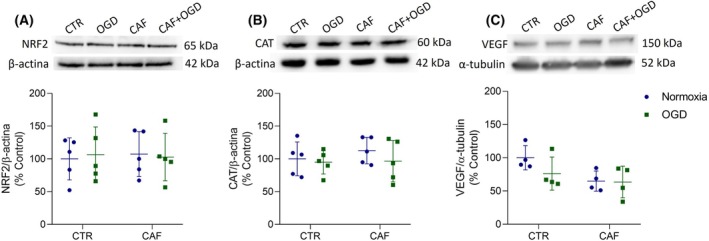
Acute caffeine exposure does not alter NRF2, catalase or VEGF protein expression during OGD. Retinas were exposed to 50 min of normoxia (●) or OGD (■) in the presence or absence of caffeine (140 μm). Protein levels were evaluated by Western blot and normalized to β‐Actin. Representative blots and densitometric analyses are shown. Values are expressed as mean ± SD (*n* = 5). (A) NRF2: Control (100.0 ± 32.03%), Caffeine (107.3 ± 34.30%), OGD (106.4 ± 42.36%), and Caffeine + OGD (102.8 ± 36.29%). Two‐way ANOVA revealed no significant effects of interaction (F(1,16) = 0.1115), treatment (F(1,16) = 0.01318), or condition (F(1,16) = 0.003433). Bonferroni's multiple comparisons test did not detect significant differences between any pair of groups. (B) Catalase (CAT): Control (100.0 ± 25.83%), Caffeine (112.6 ± 20.20%), OGD (94.94 ± 18.06%), and Caffeine + OGD (96.51 ± 31.52%). Two‐way ANOVA revealed no significant effects of interaction (F(1,16) = 0.2526), treatment (F(1,16) = 0.4184), or condition (F(1,16) = 0.9320). Bonferroni's multiple comparisons test did not detect significant differences between groups. (C) VEGF: Control (100.0 ± 18.21%, *n* = 4), Caffeine (64.93 ± 15.11%, *n* = 4), OGD (76.16 ± 24.95%, *n* = 4), and Caffeine + OGD (63.45 ± 23.74%, *n* = 4). Two‐way ANOVA revealed a significant effect of treatment (F(1,12) = 5.229, *P* = 0.0412), but no significant effects of condition or interaction. Bonferroni's multiple comparisons test did not detect significant differences between individual groups. OGD, oxygen–glucose deprivation; NRF2, nuclear factor erythroid 2–related factor 2; CAT, catalase; VEGF, vascular endothelial growth factor.

These transcriptional findings prompted us to determine whether the observed changes at the mRNA level were accompanied by corresponding alterations in protein levels. Therefore, we next evaluated NRF2 and catalase protein levels, as well as VEGF expression as an additional marker associated with ischemic stress (Fig. 4).

Quantification of NRF2 protein levels revealed no significant differences among experimental groups. Neither OGD nor caffeine treatment altered NRF2 abundance under normoxic conditions, and caffeine exposure during OGD did not modify NRF2 levels compared with OGD alone (Fig. 4A). Similarly, catalase protein content remained unchanged across conditions. No differences were detected between control and OGD groups, and caffeine treatment did not affect catalase levels in either normoxic or OGD conditions (Fig. 4B).

We next evaluated VEGF protein levels as a marker associated with ischemic signaling. None of the groups showed changes in the levels of VEGF. In particular, caffeine did not significantly modify VEGF expression under normoxic conditions nor during OGD when compared with the OGD group alone (Fig. 4C).

Collectively, although caffeine increased NRF2 mRNA expression under normoxic conditions, this transcriptional modulation was not accompanied by detectable changes in total NRF2 protein levels, nor by alterations in the protein levels of downstream antioxidant enzymes analyzed during OGD. These findings indicate that acute caffeine neuroprotection is unlikely to depend on a robust activation of the canonical NRF2‐dependent antioxidant pathway under the present experimental conditions.

**Fig. 5:**
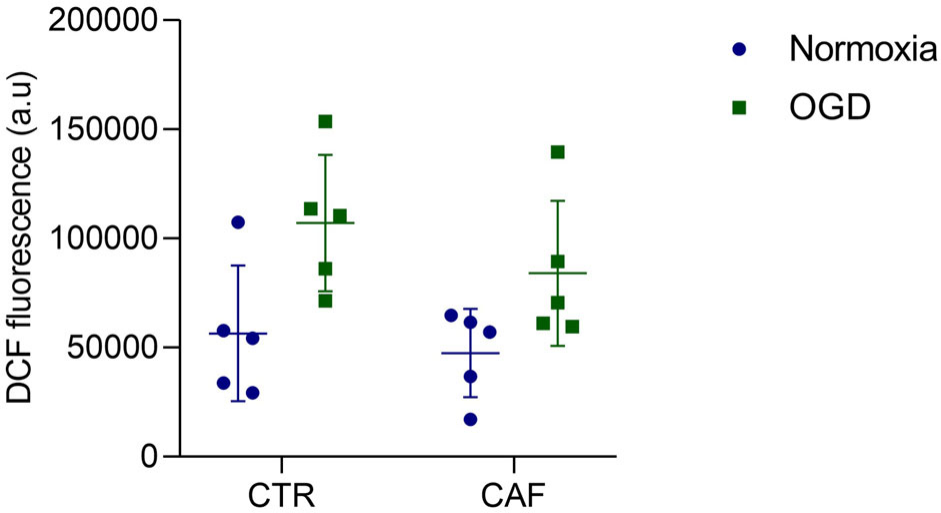
Acute caffeine does not alter OGD‐associated ROS levels measured by DCF fluorescence. Retinas were incubated with caffeine (140 μm) for 50 min of normoxia (●) or oxygen–glucose deprivation (OGD) (■). Intracellular reactive oxygen species (ROS) production was assessed using the fluorescent probe DCFH‐DA, and results are presented as DCF fluorescence (arbitrary units). Two‐way ANOVA revealed a significant effect of condition (F(1,16) = 10.97, *P* = 0.0044), with no effect of caffeine treatment (F(1,16) = 1.478, *P* = 0.2417) or interaction between factors (F(1,16) = 0.2798, *P* = 0.6041). Values are expressed as mean ± standard deviation (SD) (CTR normoxia: 56489 ± 31 076, *n* = 5; CAF normoxia: 47455 ± 20 218, *n* = 5; CTR OGD: 107015 ± 31 302, *n* = 5; CAF OGD: 84067 ± 33 247, *n* = 5). OGD, oxygen–glucose deprivation; DCF, dichlorofluorescein fluorescence.

To evaluate the putative direct effect of caffeine as an antioxidant and whether the protective effects of caffeine might be linked to an antioxidant action, we measured the oxidative stress by using the DCFDA probe (Fig. 5). Although DCF fluorescence tended to be higher under OGD conditions compared with normoxia, pairwise comparisons did not reveal significant differences between the experimental groups. In addition, caffeine treatment did not significantly modify ROS levels under either normoxic or OGD conditions, and no interaction between condition and treatment was detected (Fig. 5).

Taken together, these findings indicate that acute caffeine exposure does not significantly affect ROS levels in this model. Thus, although oxidative stress is commonly associated with ischemic injury, our results do not support a clear modulation of ROS by caffeine under the present experimental conditions.

**Fig. 6:**
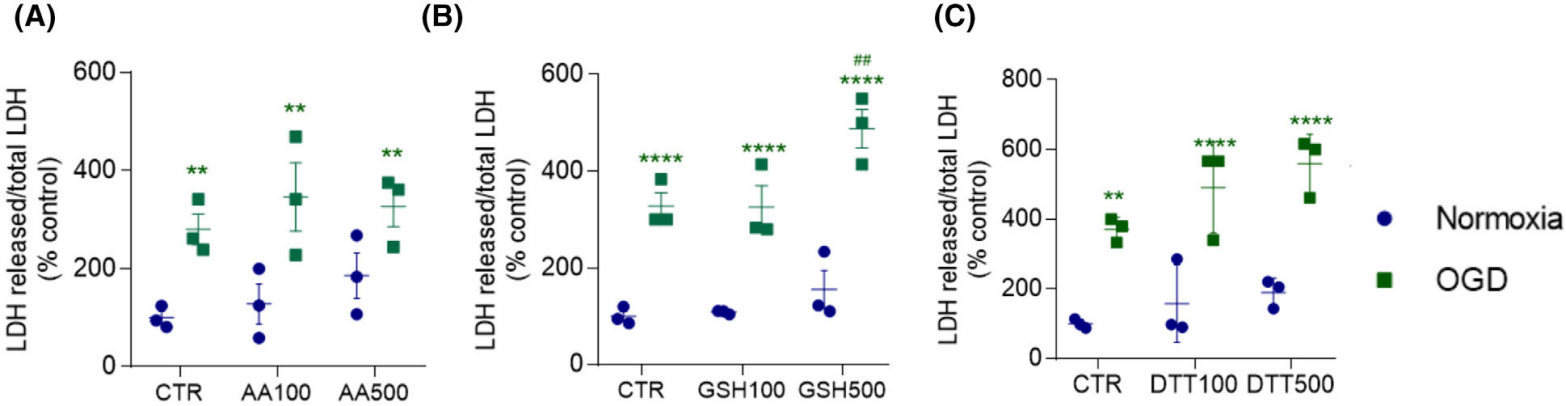
Acute incubation with antioxidant agents does not reduce OGD‐induced cell death. Retinas were exposed to 50 min of normoxia (●) or OGD (■) in the presence of ascorbic acid (AA), glutathione ethyl ester (GSH), or dithiothreitol (DTT) at two concentrations (100 μm and 500 μm). LDH release was used as an indicator of cell death. None of the antioxidants attenuated OGD‐induced LDH release. Values are expressed as mean ± SD: (A) Control (100.0 ± 22.05%, *n* = 3), AA 100 μm (127.9 ± 70.64%, *n* = 3), AA 500 μm (186.2 ± 80.29%, *n* = 3), OGD (280.7 ± 54.00%, *n* = 3), OGD + AA 100 μm (346.2 ± 120.8%, *n* = 3), OGD + AA 500 μm (327.0 ± 71.81%, *n* = 3). Two‐way ANOVA showed a significant effect of condition (F(1, 12) = 25.63, *P* = 0.0003), but no significant effects of treatment (F(2, 12) = 1.223, *P* = 0.3285) or interaction (F(2, 12) = 0.3961, *P* = 0.6814). (B) GSH – Control (100.00 ± 17.84%, *n* = 3), GSH 100 μm (108.55 ± 3.89%, *n* = 3), GSH 500 μm (155.41 ± 67.74%, *n* = 3), OGD (327.78 ± 48.11%, *n* = 3), OGD + GSH 100 μm (325.90 ± 76.58%, *n* = 3), OGD + GSH 500 μm (488.10 ± 68.64%, *n* = 3). Two‐way ANOVA showed significant effects of treatment (*P* = 0.0069) and condition (**P < 0.0001), with no significant interaction. Bonferroni's multiple comparisons indicated that OGD significantly increased LDH release vs. control (*P* = 0.0035), and OGD + GSH 500 μm further potentiated cell death compared to control (**P < 0.0001). (C) DTT – Control (100.00 ± 13.35%, *n* = 3), DTT 100 μm (158.31 ± 111.0%, *n* = 3), DTT 500 μm (189.81 ± 41.00%, *n* = 3), OGD (371.10 ± 34.21%, *n* = 3), OGD + DTT 100 μm (491.10 ± 130.9%, *n* = 3), OGD + DTT 500 μm (559.40 ± 85.16%, *n* = 3). Two‐way ANOVA indicated significant effects of treatment (*P* = 0.0345) and condition (**P < 0.0001), with no significant interaction. Bonferroni's multiple comparisons showed that OGD increased LDH release vs. control (*P* = 0.0225), and OGD combined with DTT further potentiated cell death compared to control (*P* = 0.0011–0.0002). Significant differences were also detected between normoxic and OGD conditions in the presence of DTT (DTT100/Normoxia vs. DTT100/OGD, *P* = 0.0045; DTT500/Normoxia vs. DTT500/OGD, *P* = 0.0018), as well as between OGD conditions (DTT100/OGD vs. DTT500/Normoxia, *P* = 0.0101; DTT100/Normoxia vs. DTT500/OGD, *P* = 0.0009). OGD, oxygen–glucose deprivation; LDH, lactate dehydrogenase; AA, ascorbic acid; GSH, glutathione ethyl ester; DTT, dithiothreitol.

To further investigate whether oxidative mechanisms could contribute to OGD‐induced retinal cell death, we next evaluated the effects of classical antioxidant compounds on retinal viability. To do this, we tested the effect of classic, well‐established antioxidant compounds: ascorbic acid (AA), dithiothreitol (DTT), and glutathione ethyl ester (GSH‐OEt) and measured LDH released after OGD. Surprisingly, none of these antioxidants reduced LDH release during OGD, even at concentrations typically considered more potent than caffeine in other models [24] (Fig. 6).

Given that classical antioxidant compounds failed to reproduce the protective effect of caffeine during OGD (Fig. 6), together with the results that caffeine did not modulate oxidative stress, these data suggest that caffeine antioxidant capacity may not be the primary driver of its protective effect, which may likely be mediated through alternative pathways.

**Fig. 7:**
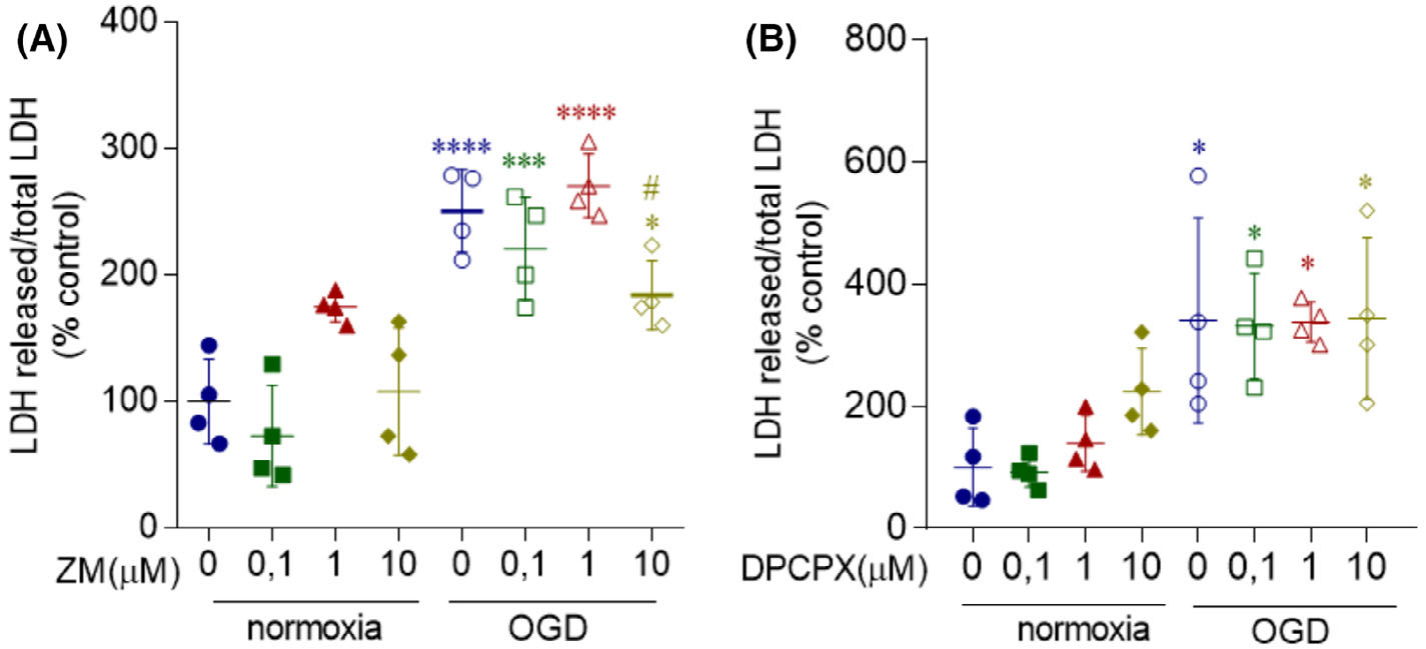
Acute blockade of A_2A_ receptors reduces OGD‐induced LDH release. Retinas were incubated with selective adenosine receptor antagonists ZM241385 (A_2A_) or DPCPX (A_1_) during 50 min of normoxia (●) or OGD (○). LDH release was measured as an indicator of cell death. For ZM241385, the values obtained were: Control (100.0 ± 33.64%, *n* = 4), ZM 0.1 μm (72.82 ± 40.04%, *n* = 4), ZM 1 μm (174.8 ± 11.58%, *n* = 4), ZM 10 μm (107.7 ± 50.33%, *n* = 4), OGD (250.6 ± 32.71%, *n* = 4), OGD + ZM 0.1 μm (220.9 ± 40.59%, *n* = 4), OGD + ZM 1 μm (270.5 ± 25.15%, *n* = 4) and OGD + ZM 10 μM (184.3 ± 27.40%, *n* = 4). Two‐way ANOVA revealed significant effects of treatment (F(3,29) = 10.54, *P* < 0.0001), condition (F(1,29) = 121.8, *P* < 0.0001) and their interaction (F(3,29) = 4.67, *P* = 0.0088). *Post hoc* Bonferroni's test showed that OGD significantly increased LDH release compared with normoxic control (*P* < 0.0001), while ZM at 1 μm under normoxia also differed from control (*P* = 0.0258). Importantly, treatment with ZM 10 μm significantly reduced OGD‐induced LDH release compared with OGD alone (*P* = 0.0073), indicating a protective effect at this concentration. In contrast, ZM 0.1 μm and 1 μm failed to prevent OGD‐induced cell death. (B) DPCPX – Control (100.0 ± 64.00%, *n* = 4), DPCPX 0.1 μm (92.65 ± 24.55%, *n* = 4), DPCPX 1 μm (138.9 ± 45.45%, *n* = 4), DPCPX 10 μm (223.9 ± 70.77%, *n* = 4), OGD (340.4 ± 168.0%, *n* = 4), OGD + DPCPX 0.1 μm (331.3 ± 86.30%, *n* = 4), OGD + DPCPX 1 μm (338.0 ± 32.62%, *n* = 4), OGD + DPCPX 10 μm (344.0 ± 132.2%, *n* = 4). Two‐way ANOVA revealed a significant effect of condition (F(1, 24) = 41.17, *P* < 0.0001), without significant treatment or interaction effects. Bonferroni's *post hoc* test indicated that OGD significantly increased LDH release compared with normoxia (*P* = 0.0207), whereas DPCPX treatment at any concentration did not reduce LDH levels. *P < 0.05, ***P < 0.001, ****P < 0.0001 vs. Control; #*P* < 0.05 vs. OGD. OGD, oxygen–glucose deprivation; LDH, lactate dehydrogenase; DPCPX, 8‐cyclopentyl‐1,3‐dipropylxanthin; ZM, ZM241385.

To better understand the mechanism behind caffeine's protective effect, we explored its action as an adenosine receptor antagonist. Specifically, we tested whether blocking A_1_ or A_2A_ adenosine receptors could mimic the effects of caffeine. Retinas were treated with selective antagonists, DPCPX (A_1_) and ZM241385 (A_2A_), during OGD. Notably, only the A_2A_ antagonist ZM241385 significantly reduced OGD‐induced LDH release to levels similar to those observed with caffeine, while A_1_ receptor blockade had no protective effect (Fig. 7). These results suggest that A_2A_ receptor antagonism may be a key contributor to caffeine's neuroprotective role in this acute model.

**Fig. 8:**
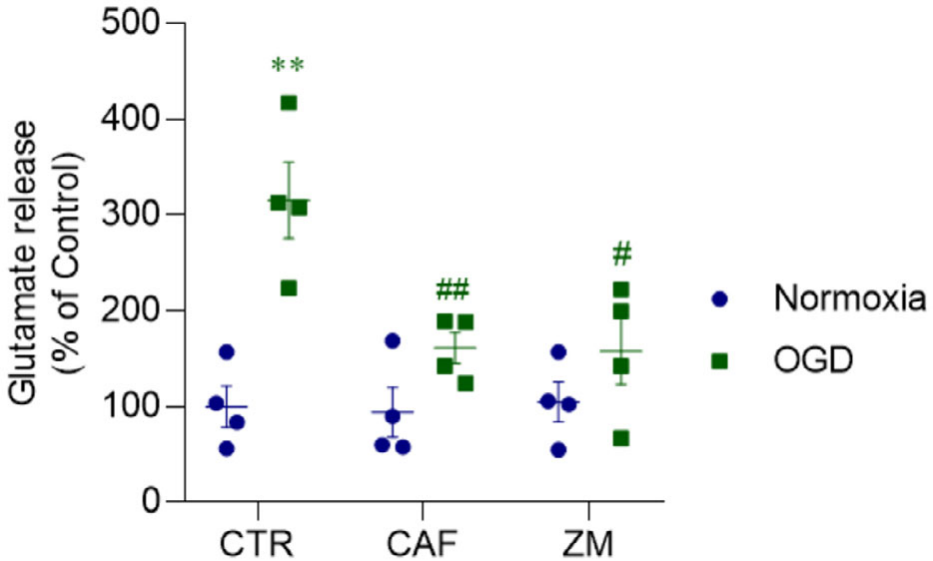
Caffeine and ZM241385 reduce OGD‐induced extracellular glutamate levels. Extracellular glutamate was quantified in E16 retinal segments subjected to 50 min of normoxia (●) or OGD (■), with or without caffeine (140 μm) or ZM241385 (10 μm). OGD markedly increased extracellular glutamate compared with normoxic controls. Caffeine and ZM241385 significantly attenuated this effect under OGD conditions, while not affecting glutamate levels in normoxia. Values are expressed as mean ± SD: Control (100.0 ± 42.59%, *n* = 4), Caffeine (93.97 ± 51.94%, *n* = 4), ZM (104.9 ± 41.73%, *n* = 4), OGD (315.4 ± 79.37%, *n* = 4), OGD + Caffeine (161.1 ± 32.74%, *n* = 4), OGD + ZM (157.8 ± 69.38%, *n* = 4). Two‐way ANOVA revealed significant effects of condition (F(1, 18) = 27.05, *P* < 0.0001), treatment (F(2, 18) = 5.905, *P* = 0.0107), and their interaction (F(2, 18) = 5.849, *P* = 0.0110). Bonferroni's *post hoc* test confirmed that OGD significantly increased glutamate compared with normoxia (***P < 0.001), and both caffeine and ZM241385 significantly reduced OGD‐induced glutamate release (**P < 0.01 vs. OGD). **P < 0.01 vs. Control; #*P* < 0.05, ##*P* < 0.01 vs. OGD. OGD, oxygen–glucose deprivation; ZM, ZM241385.

Considering the established role of glutamate excitotoxicity in ischemia‐induced neuronal damage, and the known influence of A_2A_ receptor activity on glutamate release, we next investigated whether caffeine or ZM241385 could modulate extracellular glutamate levels during OGD. As shown in Fig. 8, both caffeine and ZM treatments significantly reduced the amount of extracellular glutamate released during the insult, suggesting a common mechanism of protection via attenuation of excitotoxicity.

## Discussion

The primary question addressed in this study was whether a single, acute dose of caffeine, administered during an ischemic insult, could protect the developing retina to a similar extent as previously observed with two‐day preconditioning [17]. Our findings provide compelling evidence that acute caffeine exposure is indeed neuroprotective in this model, significantly reducing OGD‐induced retinal cell death (Fig. 1). However, this protection was not accompanied by an increase in BDNF signaling, previously implicated in longer pre‐exposure, suggesting a distinct mechanism of action for the acute effect.

The reduction in LDH release observed following caffeine administration during OGD demonstrates that even in the absence of prior sensitization, caffeine can limit ischemic damage. This result aligns with prior studies in other CNS regions showing that caffeine confers neuroprotection when administered acutely, particularly via modulation of adenosine signaling [15, 16]. Similar to our findings in the chronically treated developing retina [17], acute exposure increased phosphorylation of CREB, ERK, and AKT; these phosphorylation levels were lower in tissues subjected to OGD (i.e., OGD reduces pCREB/pERK/pAKT). However, the BDNF upregulation followed by two‐day caffeine treatment was not mimicked by acute exposure. This suggests that the acute protective effect occurs independently of the BDNF pro‐survival cascades associated with preconditioning [25]. Although we cannot rule out the involvement of the CREB, ERK, and AKT signaling pathways, the data suggest that acute exposure of caffeine protects through alternative pathways than observed in the two‐day exposure.

In line with this, an important aspect to consider is the differential involvement of BDNF in chronic versus acute caffeine exposure. While in our previous work, two‐day caffeine treatment promoted retinal protection through upregulation of BDNF signaling [17], consistent with the well‐established role of this neurotrophin in neuronal survival and plasticity [26, 27], the current data show that acute caffeine exposure does not trigger the same effect. Similar findings in other CNS models have reported that sustained caffeine exposure enhances BDNF expression and activates pro‐survival cascades [28, 29]. In contrast, the partial neuroprotection observed here (reduction in LDH release), which occurs despite OGD‐induced suppression of canonical survival pathways, appears to rely mainly on adenosine receptor modulation, occurring in the absence of a significant BDNF increase. Mechanistically, this divergence may be attributed to receptor‐specific regulation. Chronic caffeine exposure induces A_2A_ receptor downregulation while enhancing A_1_ receptor signaling [30], a process that has been associated with increased BDNF expression [31]. Conversely, during acute insult, the direct blockade of A_2A_ receptors by caffeine is sufficient to reduce glutamate release and excitotoxicity [14, 32, 33], bypassing the requirement for BDNF‐driven pro‐survival signaling. Together, these observations suggest that while BDNF is a central mediator of long‐term, preconditioning‐like effects of caffeine, its contribution is dispensable in the acute setting, where modulation of excitatory neurotransmission predominates.

Evidence from the literature suggests that caffeine may exert neuroprotective effects through antioxidant mechanisms, either by directly scavenging ROS or by enhancing endogenous antioxidant defenses [20, 21, 23]. To investigate whether such mechanisms contribute to the protective effect observed in our model, we first examined the transcriptional regulation of antioxidant pathways, focusing on the NRF2 signaling cascade. NRF2 represents a central regulator of cellular antioxidant responses and has been implicated in retinal protection in several models of ischemia and degeneration [34, 35]. In the present study, OGD induced transcriptional changes in antioxidant‐related genes such as CAT and SOD1, consistent with activation of a stress‐responsive program. However, caffeine did not restore or further enhance the expression of canonical NRF2 target genes, including CAT, SOD1, and HMOX1, during OGD (Fig. 3). These findings suggest that although oxidative stress signaling is engaged during metabolic deprivation, acute caffeine treatment does not promote a transcriptional activation of the NRF2‐dependent antioxidant machinery under these conditions.

To determine whether these transcriptional findings were accompanied by corresponding changes at the protein level, we next evaluated NRF2 and catalase abundance. Consistent with the gene expression results, no significant changes were detected in total NRF2 or catalase protein levels following caffeine exposure under either normoxic or OGD conditions (Fig. 4A,B). These observations further indicate that acute caffeine treatment does not induce a robust activation of the canonical NRF2‐dependent antioxidant pathway. It is important to note, however, that our analysis was limited to total NRF2 protein levels and did not assess its nuclear translocation, which represents the critical step required for transcriptional activation of antioxidant genes. Thus, subtle changes in NRF2 cellular localization or activity cannot be completely excluded. In addition, the protein levels of other NRF2‐regulated enzymes, such as SOD1 or HMOX1, as well as the enzymatic activity of antioxidant defenses, were not evaluated in the present study and may represent additional levels of regulation not captured in our analysis.

We also examined VEGF expression, a factor widely associated with hypoxia‐ and ischemia‐related signaling in the retina [36]. However, VEGF protein levels remained unchanged under both normoxic and OGD conditions and were not modified by caffeine treatment (Fig. 4C). Considering that the present paradigm represents a relatively short ischemic insult (50 min), it is possible that this duration is insufficient to trigger robust hypoxia‐dependent or angiogenic responses. Nevertheless, the absence of VEGF modulation further supports the interpretation that caffeine's acute neuroprotective effect is unlikely to depend on the regulation of hypoxia‐responsive or oxidative stress–related pathways.

To further evaluate whether caffeine could directly modulate oxidative stress levels during OGD, we next measured intracellular ROS production using the DCFH‐DA probe. Although OGD produced a modest overall increase in DCF fluorescence compared with normoxic conditions, caffeine treatment did not significantly alter ROS levels under either normoxia or OGD, and no interaction between condition and treatment was detected (Fig. 5). These findings indicate that, despite the well‐established role of oxidative stress in ischemic injury, acute caffeine exposure does not significantly modulate ROS levels under the present experimental conditions.

To further test whether oxidative stress contributes to OGD‐induced cell death, we examined the effects of classical antioxidant compounds. Acute administration of well‐established antioxidants, including AA, DTT, and GSH‐OEt, failed to reproduce the protective effect observed with caffeine (Fig. 6). The concentrations used were based on previous studies demonstrating robust antioxidant activity in neuronal preparations [37, 38, 39], suggesting that they were sufficient to scavenge ROS under conditions of oxidative stress. Although we cannot completely exclude the possibility that higher concentrations or longer exposure times might produce different outcomes, the absence of protection following antioxidant co‐incubation indicates that antioxidant activity alone is unlikely to explain the acute protective effect of caffeine in this model. It is also possible that the relatively short OGD duration employed here (50 min) does not generate a ROS time course that can be readily reversed by acute antioxidant intervention.

In agreement with our findings, several studies have reported that antioxidant treatments alone are insufficient to prevent excitotoxic or ischemic injury in neural tissues [4, 40], indicating that oxidative stress represents only one component of a complex injury cascade. Conversely, some reports have described the protective effects of antioxidants under OGD or ischemic conditions [41, 42], and these discrepancies may reflect differences in experimental models, tissue types, duration of the ischemic insult, or the timing of antioxidant administration relative to the injury.

Taken together, the absence of ROS modulation, the inability of classical antioxidants to reproduce caffeine's protective effect, and the lack of activation of the NRF2 antioxidant pathway collectively indicate that caffeine‐induced neuroprotection in this model is unlikely to be primarily mediated by antioxidant mechanisms.

Instead, our pharmacological experiments point to a mechanism centered on adenosine receptor antagonism. Specifically, the A_2A_ receptor antagonist ZM241385 mimicked the protective effect of caffeine, significantly reducing LDH release and extracellular glutamate accumulation during OGD (Figs 7A and 8). In contrast, the A_1_ antagonist DPCPX had no such effect (Fig. 7B). This is consistent with prior evidence that A_2A_ receptor activation enhances glutamate release and exacerbates excitotoxicity in ischemic models [12, 13, 14]. Therefore, it is plausible that caffeine's acute protective action in the retina occurs via A_2A_ receptor blockade, reducing excitotoxic neurotransmitter release during metabolic stress.

Interestingly, at first, this mechanism appears to differ from what we previously observed in our 2‐day *in vivo* caffeine exposure paradigm, where the protective effect was mimicked by the A_1_ receptor antagonist but not by A_2A_ antagonism [17]. This apparent discrepancy highlights that the contribution of adenosine receptors to caffeine's neuroprotection may vary depending on the timing and duration of exposure. The repeated, preconditioning‐like caffeine treatment increases A_1_ and decreases A_2A_ receptor [30], consistent with the observed cross‐regulation of A_1_ and A_2A_ receptors in this stage of the chick retina [43]. Therefore, the decrease seen in two‐day caffeine treatment, or the blocking of A_2A_ receptors, probably occurring with acute caffeine exposure, is in agreement with the role of this receptor in reducing neuronal excitability and promoting survival signaling. Similarly, in the acute setting, the protective effect of caffeine was also mimicked by A_2A_ receptor blockade, likely because A_2A_ activation drives glutamate release and excitotoxicity during the immediate excitotoxic conditions. Mechanistically, A_1_ and A_2A_ receptors couple to distinct G proteins and intracellular cascades: A_1_ receptors are Gi/o‐coupled and can engage PI3K–Akt survival signaling, whereas A_2A_ receptors are Gs‐coupled and favor cAMP/PKA and downstream ERK signaling that modulates neurotransmitter release. The time course of receptor modulation (chronic down−/upregulation vs. acute blockade) likely determines which downstream pathways predominate, explaining why A_1_ antagonism mimics chronic caffeine pre‐exposure while A_2A_ antagonism reproduces acute effects in our model. These differences underscore the dynamic nature of caffeine signaling in the retina and the mechanisms that act prophylactically or at the time of insult.

Taken together, our findings demonstrate that acute caffeine exposure confers significant neuroprotection to the developing retina during ischemic insult, but via alternative mechanisms from those observed with chronic treatment. Rather than engaging long‐term survival pathways, the acute effect appears to stem from modulation of purinergic signaling and inhibition of glutamate‐driven excitotoxicity. These results suggest that the timing and context of caffeine administration critically influence its neuroprotective mechanisms. Importantly, they underscore the potential of caffeine as a fast‐acting therapeutic agent in acute neurodegenerative conditions such as ischemic retinopathy or neonatal hypoxia‐ischemia, where intervention must occur during or immediately after the insult to be effective.

## Conflict of interest

The authors declare no conflict of interest.

## Author contributions

KCC, RB, and DPF had the original idea and designed the structure. KCC and RB performed the final revision. DPF, NAA, Dias, GGL, SGF, and MPR acquired the data. All authors analyzed and interpreted the data, performed a literature search, and participated in the writing of the original draft and approved the final version.

## Data Availability

The data that support the findings of this study are available in the figures and tables of this article. Additional data are available from the corresponding author upon reasonable request.
